# Geometric analysis of shape transition for two-layer carbon–silicon nanotubes

**DOI:** 10.1038/s41598-020-71026-6

**Published:** 2020-09-14

**Authors:** Xiangyan Luo, Quan Xie, Zean Tian, Xiaotian Guo, Jinmin Zhang, Tinghong Gao, Yongchao Liang

**Affiliations:** 1 Institute of Advanced Optoelectronic Materials and Technology, College of Big Data and Information Engineering, Guizhou University, Guiyang, 550025 China; 2 School of Mathematics and Physics, Anshun University, Anshun, 561000 China

**Keywords:** Materials science, Nanoscience and technology, Atomic and molecular physics

## Abstract

The two-layer nanotubes consisted of carbon atoms on the outside layer and silicon atoms on the inside layer (CNT@SiNT) show a series of diversity in the shape transitions, for instance transforming from a circle through an oval to a rectangle. In this paper, we investigate this geometric change from three perspectives. In the first aspect, we stationary time, followed by quantize in the three-dimensional Z-axis of nanotubes. In the second aspect, we stationary Z-axis, followed by quantize in the time. Finally, we tracked distance of nanotubes flattest section and roundest section. At the stationary time, the overall image of different Z-axis distance distributions is similar to a plan view of multiple ice creams, regardless of whether CNT or SiNT are on the same Z-axis, their slice plans are circle or rectangle of the projection of the Z-axis section on the XOY plane. In the stationary Z-axis, the nanotubes periodically change from a circle to an oval, and then from an oval to a rectangle at different times. Most remarkably, the distance value of deformation which we track the flattest and roundest is a constant value, and in the same distance period, there is only one roundest circle and one longest rectangle at different section and different time. The geometric analysis provided theoretical reference for the preparation of various devices and semiconductor nano-heterojunctions.

## Introduction

Since the discovery of CNTs^1^, much attention has been paid to the rich composition and structural characteristics of quasi-one-dimensional nanotubes, because it’s the basis of structure–property relation and the synthesis of novel nanotubes. Experiments have revealed that Si atoms are easily prone to *sp*^3^ hybridization while C atoms are easy to form graphite tubular *sp*^2^ hybridization, besides CNT's structures are difficult to control, especially in the chirality, and currently CNT devices can’t reach the level of silicon-based CMOS devices, and the output current and mobility of the CNT are low^2–5^. SiC block, the brittleness of bulk materials are disadvantage to its engineering application^6–9^, SiNTs are unstable^10^ . Hence, we construct the CNT@SiNT, the stability of which is probably between SiNT and CNT, and expect to find semiconductor materials which have better electrical, magnetic, and physical control performance.

Unique structuring and the quantum confinement effects of nanotubes allows it to possess peculiar electrical, optical, mechanical and magnetic quality ^11,12^, the hollow tubular structures of CNT@SiNT may have an advantage over other materials on molecular storage and transport, constrained chemical reactions, light and gas sensing. Due to CNT can be employed to solid lubricants, scanning probe tips, lithium batteries, field emission devices, or other broad application prospects fields^11–13^. The CNT@SiNT will be further researched by combining the character of CNT and SiNT.

The character and performance of nanotubes strongly depends on its structure. There are many methods to observe the internal structure of nanotubes, but most of them are statistical results, and molecular dynamics method (MD) simulation can give the details of the coordinates of each atom. However, a large number of numerical coordinates cannot accurately characterize local structure, and an effective numerical method is proposed to deeply analyze the structure and its shape transition. This paper described the shape transition of CNT@SiNT.

## Results and discussion

During MD simulation, the CNT@SiNT structure maintained periodic ripples. We observe that moving towards the top layer along the Z- axis causes periodic undulations. The fluctuation range of the ripple is also more and more regular, and finally reaches dynamic equilibrium.

**Figure 1:**
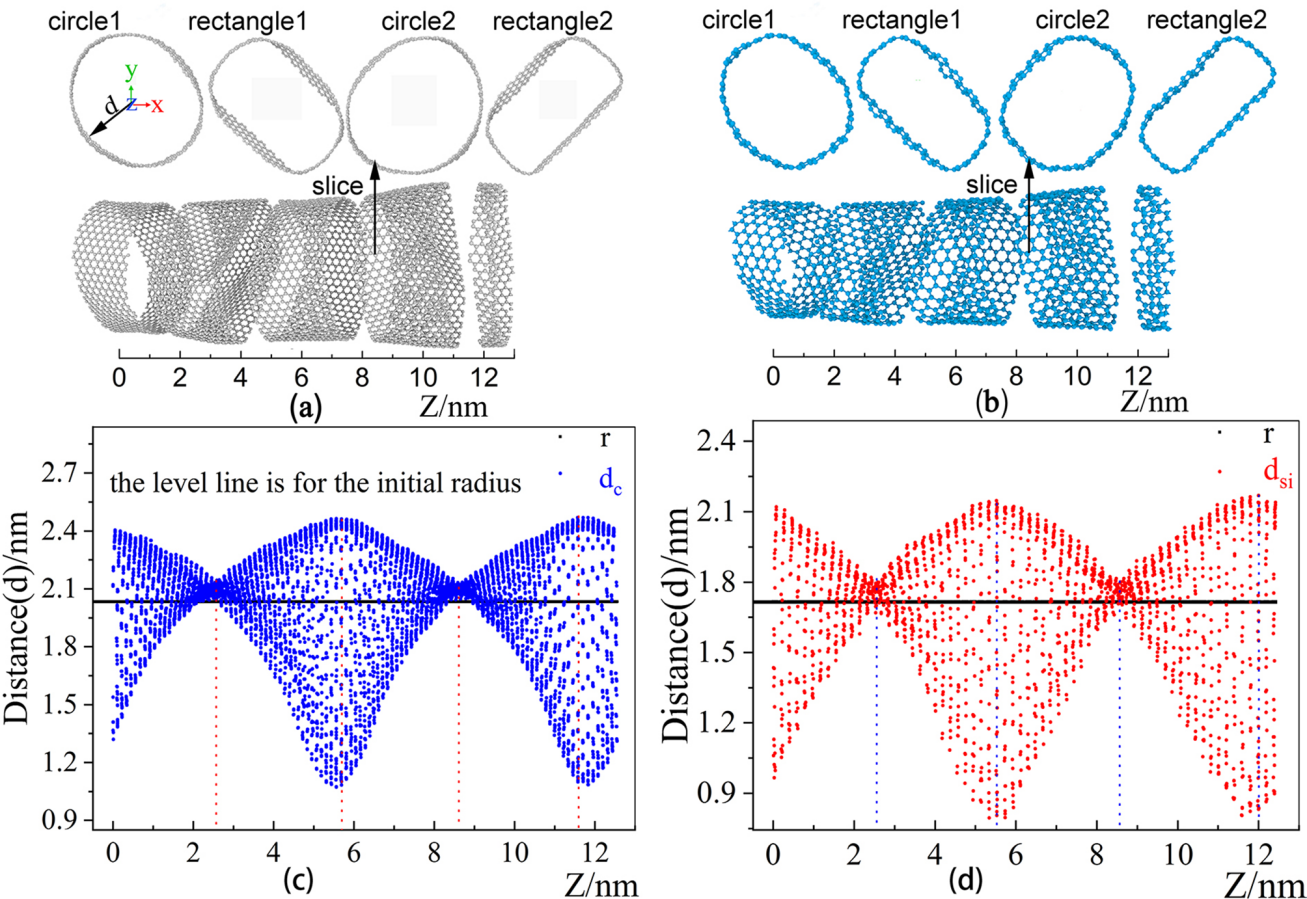
The distance distribution of all atoms from the center of the circle as the Z-axis changes. (**a**) Sliced CNT Graph, (**b**) Sliced SiNT Graph, (**c**) the distance distribution of all atoms from the center of the circle (0,0) after CNT relaxation for 0.5 ns, (**d**) the distance distribution of all atoms from the center of the circle (0,0) after relaxation of 0.5 ns for SiNT. ((**a**) and (**b**) are shown by Xiangyan Luo using python to track the flattest and roundest position after simulation and then rendered by a software in-house developed by Dr. Tian who is an author of this paper).

A large number of numerical coordinates cannot accurately characterize local structure. In order to understand the deformation situation clearly, the Z-axis distance distribution corresponding to all the atoms at 0.5 ns is shown in Fig. 1. The Z-axis distance distribution of the XOY plane of CNT and SiNT is shown in Fig. 1c and d. The horizontal black line represents the initial standard model nanotube, and its value is the half value of d in diameter. The distance distribution formula for the ice cream of the entire distribution image arrangement is given by

1 $$ dzi = \sqrt {x^{2} + y^{2} } $$

The distance distribution map has two particularly concentrated distances and two particularly scattered distances. The nanotubes are sliced along the Z axis. The cut points are Z1 = 2.3 nm, Z2 = 5.5 nm, Z3 = 8.5 nm and Z4 = 11.5 nm, the slice wide is 0.78 nm, and the slice diagrams are shown in Fig. 1a and b. As shown in Fig. 1a and c, it can be seen from that after relaxation of 0.5 ns, the flattest deformation of CNT are at 5.7 nm and 11.6 nm, which is the most intense change of nanotubes, at 2.4 nm and 8.6 nm which place is the roundest. As can be seen from Fig. 1b and d, the flattest deformation of SiNT are at near 5 nm and 12 nm after 0.5 ns. at 2.5 nm and 8.6 nm which place is the roundest. Above all, it can be concluded that the flattest and roundest deformation of the inner and outer layers located at the same position and time, and different Z-axis change periodically with the Z-axis at the same time.

**Figure 2:**
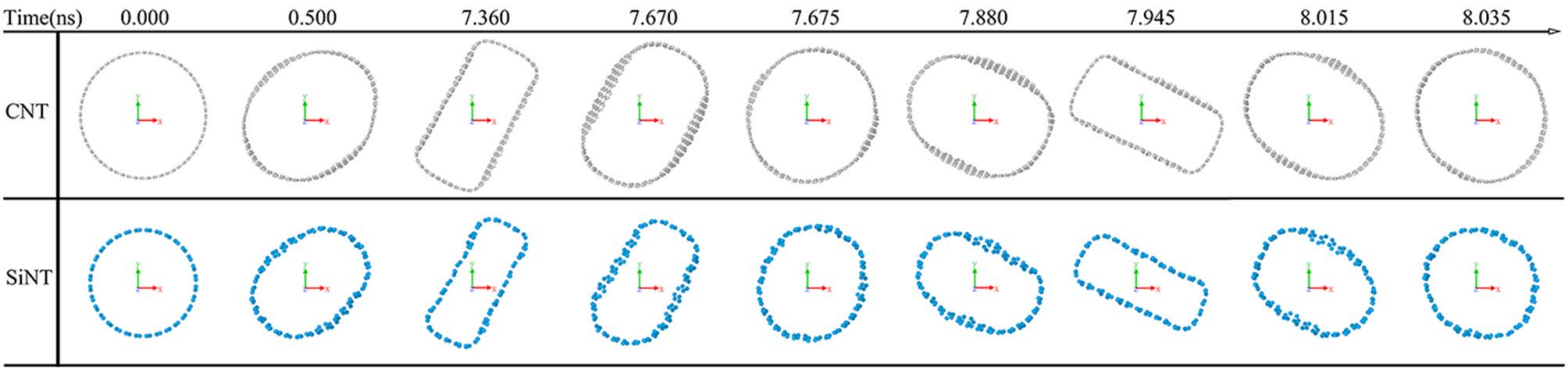
CNT @ SiNT in the plane of Z = 2.3 nm, the deformation of nanotubes vary with time, where light blue is for Si atoms and light gray for C atoms.

Nanotubes vibrating back and forth over time is sliced at the plane of Z = 2.3 nm and displayed at different times as shown in Fig. 2. It was found that the nanotubes repeatedly changed from a circle to an oval, from an oval to a rectangle, from a rectangle to an oval and from an oval to a circle over time at the plane of Z = 2.3 nm. Sometimes, when it is the roundest, it will suddenly return to the rectangle.

**Figure 3:**
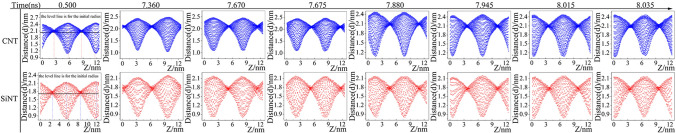
CNT and SiNT Static distance distribution of the Z-axis corresponding to the deformation of nanotubes vary with time, where blue scatter is for d_c_ and red for d_si_.

As shown in Fig. 3, a static distance distribution corresponding to the Z-axis every deformation limitation starting from 0.5 ns indicates that the circular and rectangular points move periodically on the Z-axis, and the corresponding distance values do not fluctuate apparently (is always confined in a certain range).

**Figure 4:**
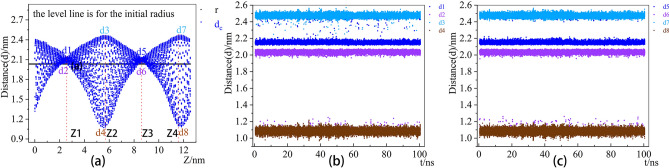
the tracked data of CNT circular section and rectangular section.

Furthermore, their distance distribution values were found to be constant over time by slicing and tracking the CNT circular and rectangular section. From Fig. 4 and Table 1, the distance distribution data elicit *d*_1_≈*d*_5_≈2.15 nm, *d*_2_≈*d*_6_≈2.03 nm, *d*_3_≈*d*_7_≈2.47 nm, and *d*_4_≈*d*_8_≈1.08 nm, which correspond with Fig. 4b and c. The error value of this fitting does not exceed 6.2%. Compared with the distance of standard CNT to *d*_initial_ = 2.0340 nm, the flattened nanotubes could not be recovered into standard nanotubes.

**Table 1:** Data distribution description table of CNT nanotubes.

Tracking point	mean	std	min	25%	50%	75%	max
d1	2.152896	0.029656	2.114550	2.141438	2.149275	2.158390	2.503420
d2	2.034929	0.053972	1.100310	2.029110	2.039390	2.048480	2.075880
z1	3.302185	1.768200	0.161730	1.796493	3.290993	4.805614	7.012425
d3	2.474295	0.020223	2.240490	2.461498	2.473950	2.487073	2.569730
d4	1.083733	0.026761	0.968080	1.065890	1.083605	1.102050	1.217230
z2	3.195269	1.715516	0.157730	1.713334	3.189825	4.643060	6.987940
d5	2.151945	0.024761	2.113020	2.141380	2.149290	2.158240	2.494460
d6	2.033599	0.062201	1.108220	2.028780	2.039320	2.048250	2.076140
z3	9.520221	1.791063	3.155480	7.991434	9.497415	11.05845	12.53710
d7	2.474662	0.019030	2.405270	2.461480	2.474120	2.487203	2.563300
d8	1.083818	0.026938	0.970040	1.065720	1.083655	1.102163	1.187310
z4	9.410896	1.746298	1.171125	7.935365	9.372108	10.89149	12.54240

**Figure 5:**
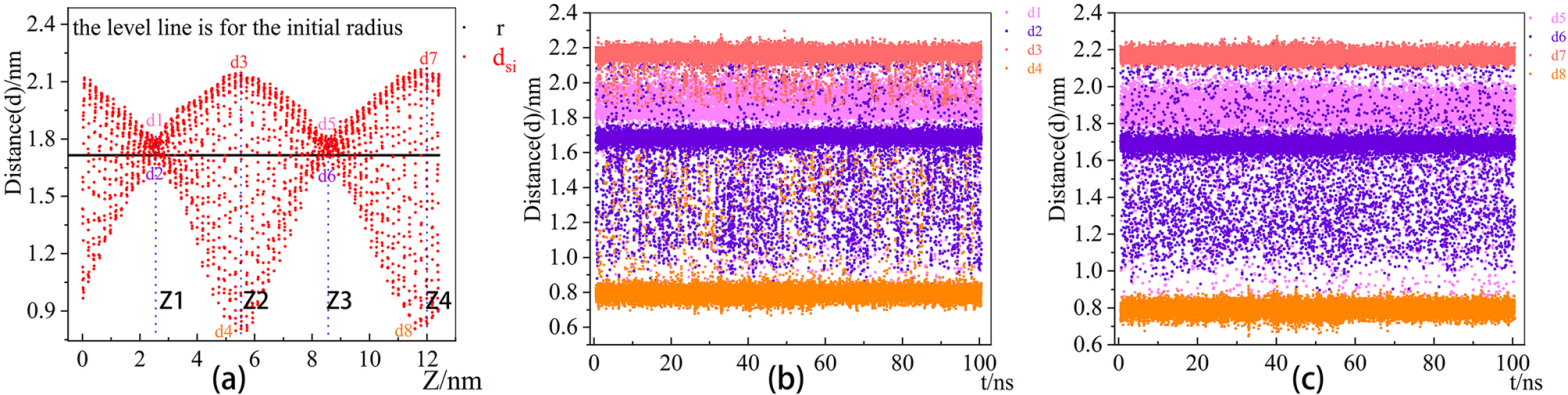
the tracked data of SiNT circular section and rectangular section.

From Fig. 5 and Table 2, the distance distribution data reveal d1≈d5≈1.82 nm, d2≈d6≈1.62 nm, d3≈d7≈2.16 nm, and d4≈d8≈0.80 nm, which correspond with Fig. 5b and c. The error value of this fitting does not exceed 16.0%. Compared with the distance of standard SiNT to d_initial_ = 1.6936 nm, the flattened nanotubes could not be recovered into standard nanotubes.

**Table 2:** Data distribution description table of CNT nanotubes.

Tracking point	mean	std	min	25%	50%	75%	max
d1	1.829673	0.159979	0.73392	1.813960	1.833905	1.907480	2.168690
d2	1.619443	0.193196	0.84772	1.644900	1.680830	1.700460	2.204510
z1	1.784160	1.012342	0.15646	0.996888	1.656688	2.465274	6.373705
d3	2.155985	0.051466	1.86249	2.145350	2.164670	2.183340	2.295540
d4	0.816478	0.121099	0.66207	0.772300	0.793560	0.816470	1.622850
z2	2.794657	1.563524	0.15727	1.637030	2.642510	3.741085	7.097285
d5	1.820491	0.173492	0.74678	1.810960	1.836235	1.912338	2.159440
d6	1.630567	0.188276	0.89625	1.647768	1.681880	1.702380	2.233140
z3	5.247539	1.486582	1.33205	4.084508	5.175795	6.328755	11.855000
d7	2.167852	0.025343	2.02738	2.150440	2.167285	2.184860	2.272360
d8	0.791807	0.031318	0.64743	0.771120	0.792155	0.813440	0.918440
z4	8.799695	1.723770	1.61383	7.530784	8.660618	9.911930	12.504600

**Figure 6:**
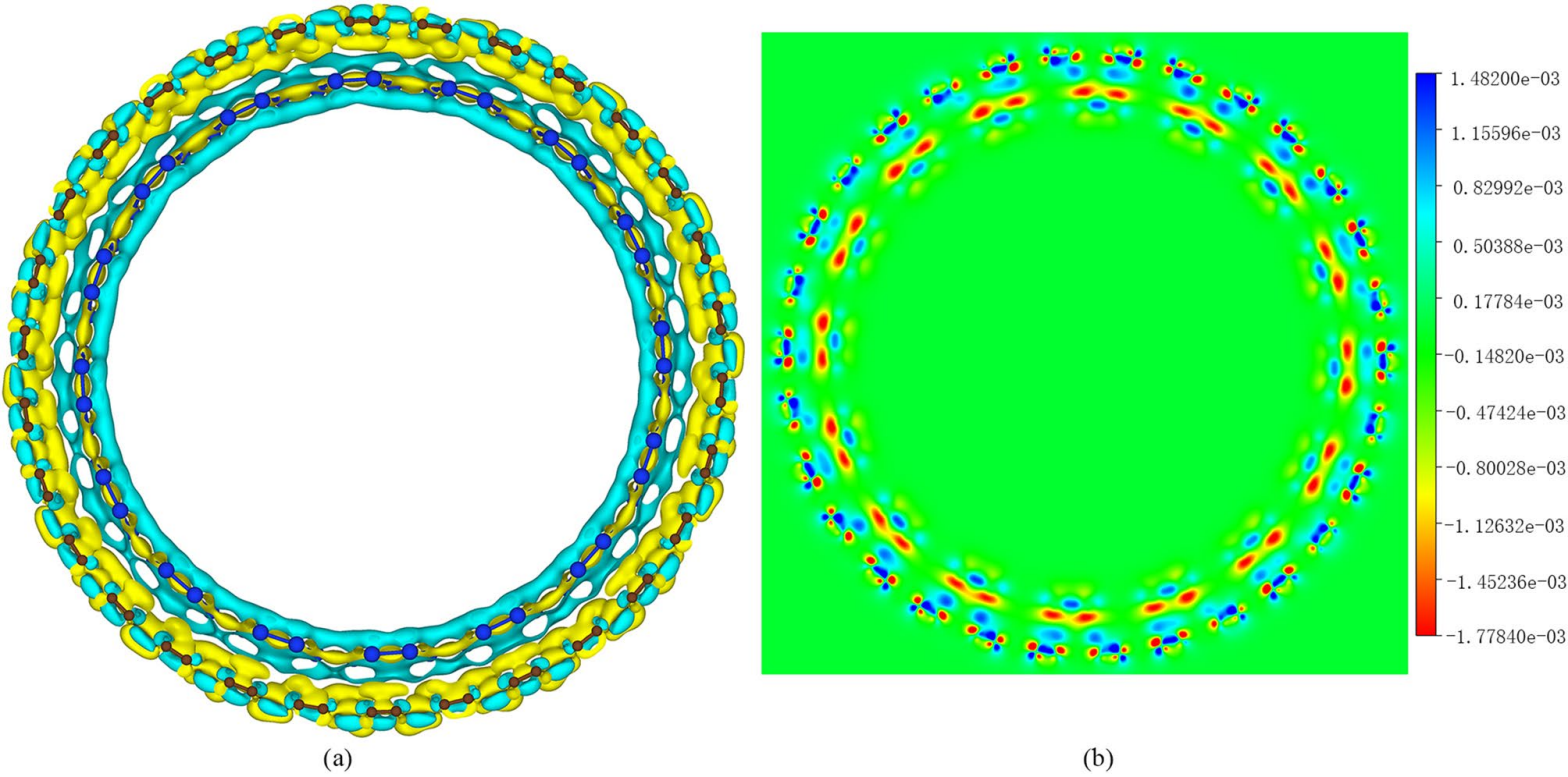
the difference electron density of SiNT@CNT in 3D (**a**) and 2D (**b**) model. (was generated using the software of VESTA^14^).

Based on the above tracking and statistical analysis, a physical level analysis will be discussed following. The reason of deformation transformation is caused by compression and bending of the nanotube under pressure driven competition. When CNT@SiNT shrink in radius, it will be deformed and its curvature is increased. Besides, shrink of CNT@SiNT weaken its compressive strain energy, reduce the area of section and diminish its bending strain energy. As shown in Fig. 6 in the revised version, the extended calculation on charge transfer in SiNT@CNT revealed the electrons transferred from the outermost wall to the inner one. There is a clear overlap of electron clouds between the two atomic layers, thus there exists interaction between the carbon and silicon layers.

Analyze and summarize, the CNT@SiNT show a series of diversity in the shape transitions with 0 Gpa, such as transforming a circle to an oval or from an oval to a rectangle. Since the reduction in cross-sectional area reaches the limitation and no longer compresses, its rectangle deformation remains unchanged at that moment. Then, it suddenly turns back into a circle or changes direction to return back to the oval under other forces. From the circle to the oval and then from the oval to the rectangle, the energy dissipation of conversion makes the nanotubes couldn’t recover back to the level of model nanotube which is the roundest section. This is the reason that nanotubes are curved and unsmoothed we observed in the experiments^15^. We deduce from the deformation transition that there exists strong interaction between C atoms and Si atoms.

## Mathematic model

**Figure 7:**
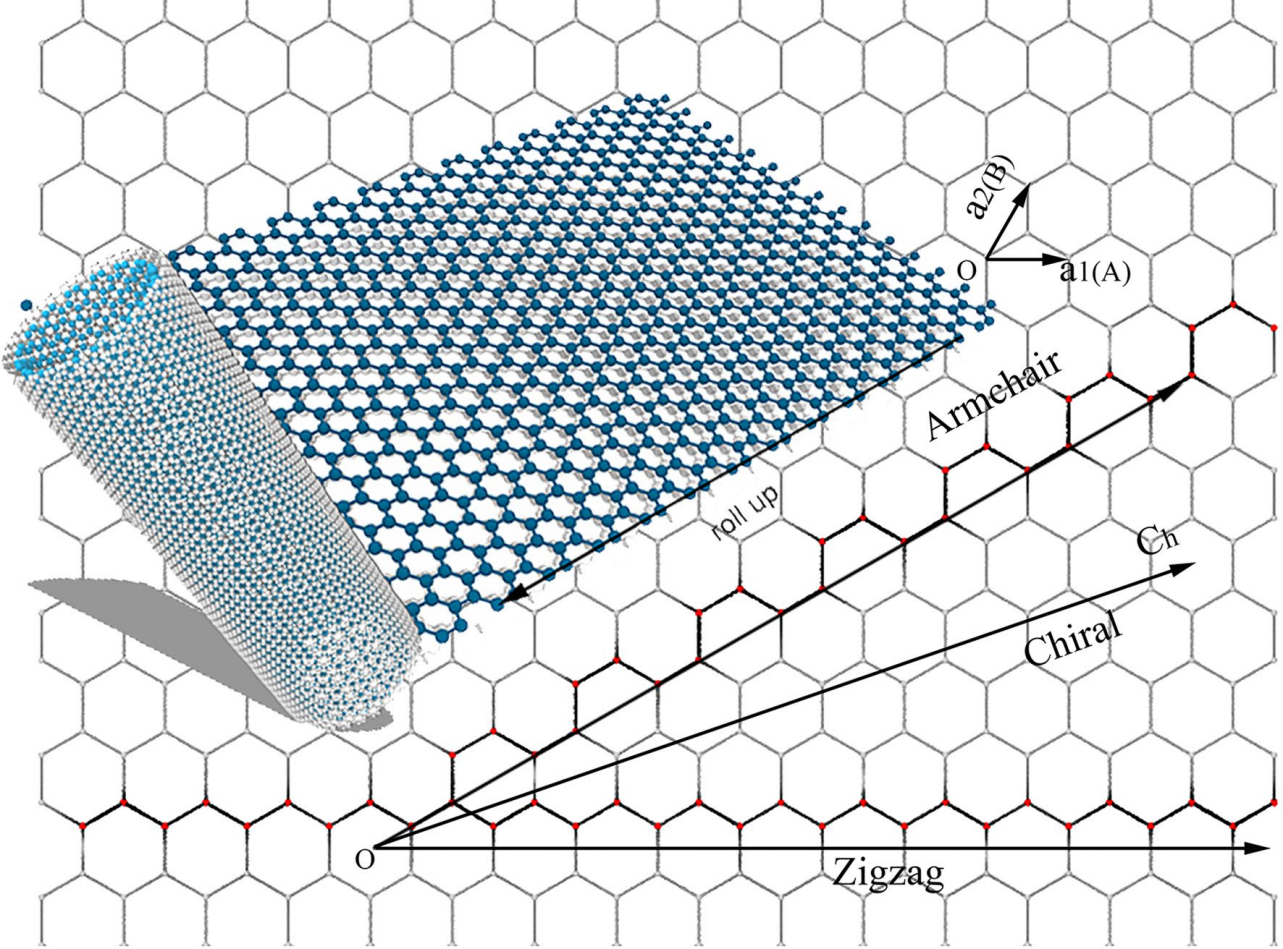
Graphene curls into nanotubes, where blue balls are for Si atoms and gray for C atoms. (is the model coordinates generated by Xiangyan Luo using python and then rendered by a software in-house developed by Dr. Tian who is an author of this paper. Finally, with Photoshop the three pictures (the honeycomb grid, the graphene sheet in 3D model, and the two-layer nano-tube) are combined and polished).

In Fig. 7, we constructed two-layer nanotubes with carbon (C) atoms outside and silicon (Si) atoms inside, the two layers are parallel and perpendicularly oriented according to the following modeling method, which is obtained by improving C. T. White's method^16^. The parameters of the models are shown in Table 3.

**Table 3:** Model parameters.

Element	n	m	Bond_length (nm)	N_periodicity	Diameter (nm)	Radius/d_initial_ (nm)	Length (nm)	Atoms_number
C	30	30	0.1421	51	4.0680	2.0340	12.5435	6120
Si	16	16	0.2245	32	3.3871	1.6936	12.2877	2048

## Simulation method

Molecular dynamics (MD) method simulations were carried out with LAMMPS and results relied on the standard empirical Tersoff potential of CNT@SiNT^3,4^. The energy E, as a function of the atomic coordinates is taken to be

3 $$ \begin{aligned} E & = \sum\limits_{i} {E_{i} } = \frac{1}{2}\sum\limits_{i \ne j} {V_{ij} } \\ V_{ij} & = f_{C} (r_{ij} )\left[ {f_{R} (r_{ij} ) + b_{ij} f_{A} (r_{ij} )} \right] \\ f_{R} (r_{ij} ) & = A_{ij} \exp ( - \lambda_{ij} r_{ij} ) \\ f_{A} (r_{ij} ) & = - B_{ij} \exp ( - \mu_{ij} r_{ij} ) \\ \end{aligned} $$

4 $$ \begin{aligned}    & f_{C} (r_{{ij}} ) = \left\{ {\begin{array}{*{20}l}    {1,} \hfill & {r_{{ij}}  < R_{{ij}} } \hfill  \\    {\frac{1}{2} + \frac{1}{2}\cos [\pi (r_{{ij}}  - R_{{ij}} )/(S_{{ij}}  - R_{{ij}} )],} \hfill & {R_{{ij}}  < r_{{ij}}  < S_{{ij}} } \hfill  \\    {0,} \hfill & {r_{{ij}}  > S_{{ij}} } \hfill  \\   \end{array} } \right. \\     & b_{{ij}}  = \chi _{{ij}} (1 + \beta _{i}^{{n_{i} }} \zeta _{{ij}}^{{n_{i} }} )^{{ - 1/2n_{i} }} ,\zeta _{{ij}}  = \sum\limits_{{k \ne i,j}} {f_{C} (r_{{ik}} )} \omega _{{ik}} g(\theta _{{ijk}} ), \\     & g(\theta _{{ijk}} ) = 1 + c_{i}^{2} /d_{i}^{2}  - c_{i}^{2} /[d_{i}^{2}  + (h_{i}  - \cos \theta _{{ijk}} )^{2} ], \\     & \lambda _{{ij}}  = (\lambda _{i}  + \lambda _{j} )/2 \\     & \mu _{{ij}}  = (\mu _{i}  + \mu _{j} )/2 \\     & A_{{ij}}  = (A_{i} A_{j} )^{{1/2}}  \\     & B_{{ij}}  = (B_{i} B_{j} )^{{1/2}}  \\     & R_{{ij}}  = (R_{i} R_{j} )^{{1/2}}  \\     & S_{{ij}}  = (S_{i} S_{j} )^{{1/2}}  \\  \end{aligned}  $$

Here *i*, *j*, and *k* label the atoms of the system, *r*_*ij*_ is the length of the *ij* bond, and *θ*_*ijk*_ is the bond angle between bonds *ij* and *ik*.

MD simulation was carried out in cubic box under the periodic boundary condition that the axial (Z) length of two layers of nanotubes was approximately equal. Under 0 Gpa pressure, in order to stabilize the CNT@SiNT, a Nose–Hoover thermostat was initially used to balance the cylindrical CNT@SiNT at 300 K. Nanotubes have been placed in the NVE integrated environment to perform MD relaxation simulation running 2.01 × 10^8^ steps, which took 100.5 ns. The simulation parameters are shown in Table 4.

**Table 4:** Simulation parameters.

Boundary conditions	ffp
Temperature	Isothermal 300 k
Pressure	0 Gpa
Time/ step	0.5 fs/step
Ensemble	*NVE*

LDA calculations were performed both with a plane wave and a localized basis set package^17^. In the VASP calculations the projector augmented wave method was applied using a 400 eV plane wave cutoff energy. For the SiNT@CNT, less K points were used. Since each SiNT@CNT unit cell contains 184 atoms in the unit cell, we calculated for one unit cell. As these codes use periodic boundary conditions, only commensurate SiNT@CNT can be studied by them in practice. Otherwise, a model of incommensurate nanotubes would require huge supercells.

## Conclusions

In conclusion, the distance distribution constants can not only characterize the structural deformation amplitude of CNT@SiNT, but also characterize other structural deformation amplitude of tube structure. Furthermore, we expect similar shape transitions to appear in three dimensions tubular structure, such as nanotubes of other elements. All these physical objects are considered deformable, and the limit value of deformability is universal.
